# *In silico* analysis of TRPM4 variants of unknown clinical significance

**DOI:** 10.1371/journal.pone.0295974

**Published:** 2023-12-15

**Authors:** Svetlana I. Tarnovskaya, Anna A. Kostareva, Boris S. Zhorov

**Affiliations:** 1 Almazov National Medical Research Centre, St. Petersburg, Russia; 2 Sechenov Institute of Evolutionary Physiology & Biochemistry, Russian Academy of Sciences, St. Petersburg, Russia; 3 Department of Women’s and Children’s Health and Center for Molecular Medicine, Karolinska Institutet, Stockholm, Sweden; 4 Department of Biochemistry and Biomedical Sciences, McMaster University, Hamilton, Canada; Human Genetics and Genome Research Institute, National Research Centre, EGYPT

## Abstract

**Background:**

TRPM4 is a calcium-activated channel that selectively permeates monovalent cations. Genetic variants of the channel in cardiomyocytes are associated with various heart disorders, such as progressive familial heart block and Brugada syndrome. About97% of all known TRPM4 missense variants are classified as variants of unknown clinical significance (VUSs). The very large number of VUSs is a serious problem in diagnostics and treatment of inherited heart diseases.

**Methods and results:**

We collected 233 benign or pathogenic missense variants in the superfamily of TRP channels from databases ClinVar, Humsavar and Ensembl Variation to compare performance of 22 algorithms that predict damaging variants. We found that ClinPred is the best-performing tool for TRP channels. We also used the paralogue annotation method to identify disease variants across the TRP family. In the set of 565 VUSs of hTRPM4, ClinPred predicted pathogenicity of 299 variants. Among these, 12 variants are also categorized as LP/P variants in at least one paralogue of hTRPM4. We further used the cryo-EM structure of hTRPM4 to find scores of contact pairs between parental (wild type) residues of VUSs for which ClinPred predicts a high probability of pathogenicity of variants for both contact partners. We propose that 68 respective missense VUSs are also likely pathogenic variants.

**Conclusions:**

ClinPred outperformed other in-silico tools in predicting damaging variants of TRP channels. ClinPred, the paralogue annotation method, and analysis of residue contacts the hTRPM4 cryo-EM structure collectively suggest pathogenicity of 80 TRPM4 VUSs.

## 1. Introduction

The transient receptor potential channel TRPM4 (member 4 of the melastatin subfamily) is a calcium-activated ion channel that selectively permeates monovalent cations. The channel is widely distributed in various organs. In the myocardial tissue, TRPM4 is involved in cardiac conduction, pacing, the action potential repolarization and other processes. Human TRPM4 (hTRPM4) is among the most important cardiac TRP channels whose pathogenic variants are associated with cardiac arrhythmias [1, 2]. Mutations in the hTRPM4 gene result in progressive familial heart block type I (PFHBI), bundle-branch block (BBB), right bundle branch block, isolated cardiac conduction disease (ICCD) and Brugada syndrome [3–6]. As of November 2023, the ClinVar database [7] lists 688 missense variants of hTRPM4. Among these, 633 variants are of unknown clinical significance (VUS), 31 variants have conflicting interpretation of pathogenicity (CIP), four variants are described as likely pathogenic or pathogenic (LP/P), and 20 variants are characterized as likely benign or benign (LB/B). The American College of Medical Genetics and Genomics and the Association for Molecular Pathology (ACMG/AMP) recommend *in silico* predictive algorithms for variants’ interpretation [8]. A large number of VUSs in hTRPM4 motivates employing bioinformatics to predict impact of missense variants on the channel function and distinguish those variants that may be clinically relevant.

Numerous variant interpretation tools based on different principles have been developed to predict pathogenicity and tolerance of genetic variants [9]. The success rate of these tools varies from 60 to 80% [8]. Each tool and its underlying algorithm have strengths and weaknesses. The ACMG/AMP guideline recommendsusing multiple software approaches for variant interpretation without specifying the number or types of algorithms. The choice of bioinformatics tools is critical for correct variant interpretation. Choosing the best-performing tool and pathogenicity threshold for a specific protein family increases reliability of pathogenicity predictions.

The performance of *in silico* tools may depend on the disease phenotype [10]. For instance, tools MetaLR, MetaSVM, and MCap demonstrated the top performance in predicting pathogenicity for variants associated with abnormalities in the cardiovascular system [10]. However, some methods yielded many false-positive and false-negative predictions of pathogenicity in individual protein families [8, 9, 11, 12]. For example, MetaSVM predicted a pathogenic effect for 75% of benign variants of the cardiac sodium channel Nav1.5 [11]. Choosing a tool with a high success rate of correct predictions for specific protein families and adjusting the pathogenicity threshold allows to improve predictions [10, 11].

Earlier we applied bioinformatics tools combined with the paralogue annotation method [13] to reclassify as LP/P variants numerous VUSs of sodium channel Nav1.5 and calcium channel Cav1.2 [11, 12]. The paralogue annotation method employs a multiple sequence alignment of functionally and structurally related proteins and focuses on residues in sequentially matching positions where a disease mutation is known for at least one family member. Then a VUS in the matching position of the channel under investigation is assumed to be a LP/P variant [11, 14].

The TRPM4 channel belongs to the superfamily of transient receptor potential (TRP) channels. Some members of this family have attracted increasing attention in the past decade as promising drug targets for treatment of cardiovascular diseases [15], neurodegenerative disorders [14], inflammation [16], and Type II diabetes [17]. Information about pathogenic variants of these paralogues may be useful for interpretation of uncharacterized hTRPM4 VUSs.

Here, we composed a large dataset to test performance of various predictors in identifying known LP/P and benign variants in the superfamily of TRP channels. We collected LP/P missense variants from three databases listed in section 2.2 and benign missense variants from the gnomAD database. We evaluated the performance of 22 popular bioinformatics prediction tools and found that ClinPred outperformed other tools for the superfamily of TRP channels. ClinPred and the paralogue annotation method consensually predicted that 12 VUSs of hTRPM4 may be damaging variants. We further employed the cryo-EM structure of the hTRPM4 channel [18] to find scores of contact pairs between parental (wild type) residues of likely pathogenic missense VUSs’ (according to ClinPred results for both contact partners) and proposed that 68 respective VUSs can be damaging variants. We propose that 80 missense VUSs, which are described in this study, may be associated with hTRPM4 dysfunctions.

## 2. Methods

### 2.1. Sequence data of human channels

The hTRPM4 amino acid sequence was obtained from the UniProt database, entry Q8TD43 [19]. For the paralogue annotation method, we have chosen proteins from the following subfamilies of human TRP channels: TRPC (Canonical), TRPV (Vanilloid), TRPM (Melastatin), TRPP (Polycystin), TRPML (Mucolipin), and TRPA (Ankyrin).UniProt IDs for the proteins used in the analysis are shown in Table 1.

**Table 1:** Human channels in the TRP superfamily.

Family	UniProt ID	Family	UniProt ID	Family	UniProt ID	Family	UniProt ID
TRPA1	O75762	TRPC5	Q9UL62	TRPM5	Q9NZQ8	TRPV1	Q8NER1
TRPP2	Q13563	TRPC6	Q9Y210	TRPM6	Q9BX84	TRPV2	Q9Y5S1
TRPP3	Q9P0L9	TRPC7	Q9HCX4	TRPM7	Q96QT4	TRPV3	Q8NET8
TRPP5	Q9NZM6	TRPM1	Q7Z4N2	TRPM8	Q7Z2W7	TRPV4	Q9HBA0
TRPC1	P48995	TRPM2	O94759	TRPML1	Q9GZU1	TRPV5	Q9NQA5
TRPC3	Q13507	TRPM3	Q9HCF6	TRPML2	Q8IZK6	TRPV6	Q8TDD5
TRPC4	Q9UBN4	TRPM4	Q8TD43	TRPML3	Q8TDD5		

UniProt ID: accession number of a protein in the UniprotKB database.

Family: gene name of a human TRP channel.

### 2.2. Collection of variants

LP/P variants for hTRPM4 and its paralogues (Table 2) were collected from three databases: Humsavar (https://www.uniprot.org/docs/humsavar, updated 1March 2023)), Ensembl Variation [20] (updated 2023-03-01) and ClinVar [7] (updated 2023-04-10). Only likely pathogenic and pathogenic variants (*LP/P)* were extracted from databases Ensembl Variation and Humsavar. From the ClinVar database, we selected those variants, which are characterized as *‘pathogenic’* or *‘likely pathogenic’* and are associated with specific clinical conditions. Moreover, we excluded variants from pathogenic dataset which were characterized as ‘LB/B’ or ‘VUS’ in ClinVar. VUSs were extracted from the ClinVar database where field ‘*Clinical Significance*’ has words ‘*Uncertain significance*’. Benign (neutral) variants along with their minor allele frequencies (AF) were obtained from the population database gnomAD [21]. Variants with AF > 0.00005, which are absent in ClinVar, were considered benign [22, 23]. The number of collected LP/P, VUS, and benign variants is shown in Table 2. All variants were combined in one broad dataset (S1 Table).

**Table 2:** Known variants in the hTRPM4 channel and its paralogues.

Gene ^a^	Uniprot ID^b^	LP/P ^c^	VUS ^d^	Benign^e^
TRPA1	O75762	1	31	36
TRPC6	Q9Y210	17	90	10
TRPM1	Q7Z4N2	22	18	4
TRPM4	Q8TD43	10	565	6
TRPM6	Q9BX84	1	7	3
TRPM7	Q96QT4	1	29	42
TRPV4	Q9HBA0	72	349	0
TRPV6	Q9H1D0	6	28	2

^a^ Genes of the TRP-superfamily, which contain one or more LP/P variant in public databases

^b^ Protein index in pathogenic variants

^c^ LP/P is Likely Pathogenic/Pathogenic variant

^d^ Variants of unknown clinical significance

^e^ Variant from the gnomAD database, which occurs in a population with allele frequency >0.00005 and are absent in the ClinVar database.

### 2.3. Topology of the TRPM4 channel

**Fig 1:**
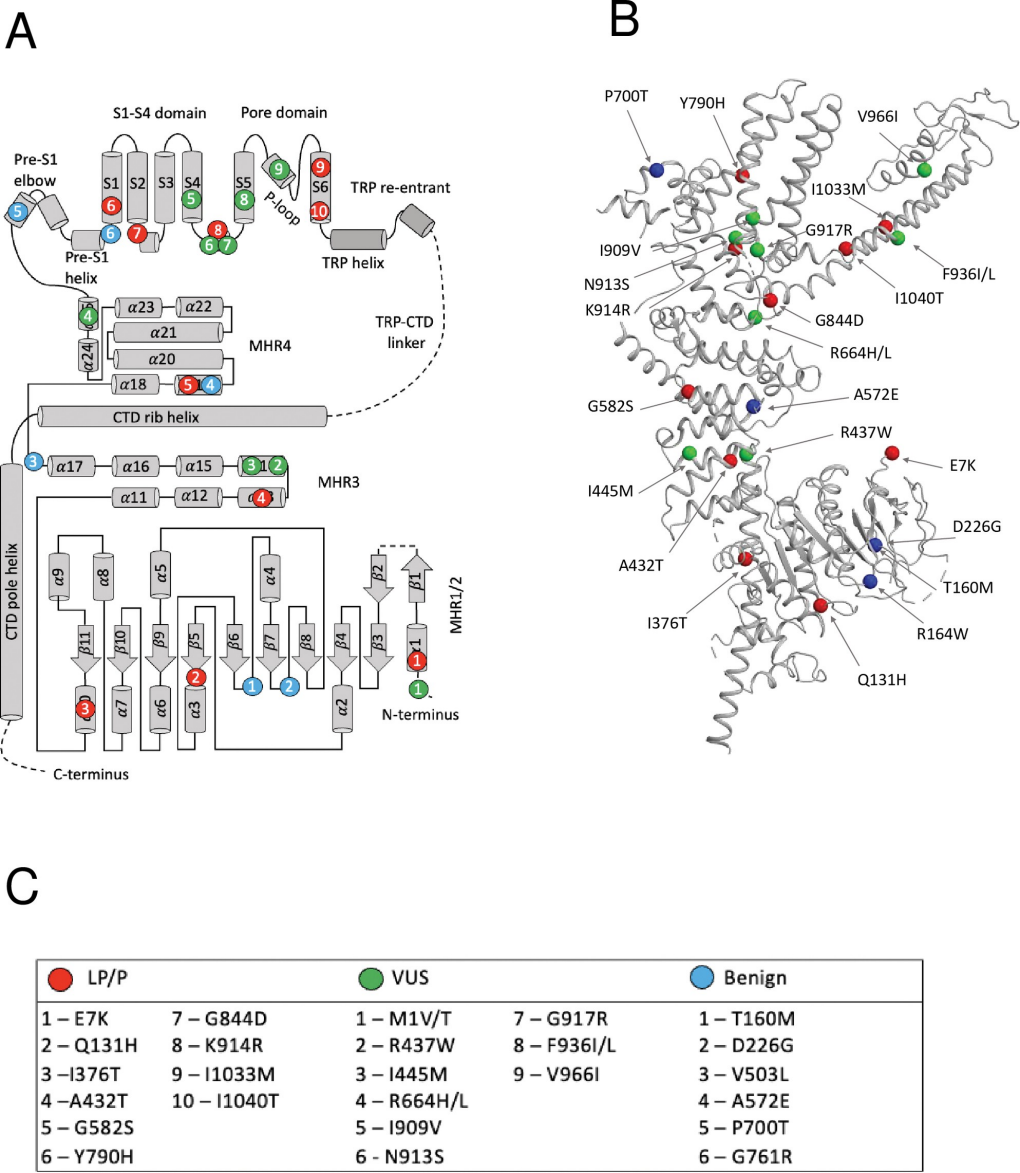
A subunit of hTRPM4. (A) The N-terminus has four melastatin regions (MHR1-4) and Pre-S1 region. The transmembrane part contains six helices (S1-S6). Modified from [18]. Helices S5, S6 and P-loop contribute a quarter to the pore module. The C-terminus contains TRP and CTD helices. Dashed lines indicate regions, which are not resolved in the cryo-EM structure [18]. (B) 3D structure (PDB ID: 5WP6) [18]. LP/P, benign and VUS variants with high ClinPred and paralogue annotation scores are red, blue and green spheres, respectively. (C) List residues shown in B.

Domain organization and topology of the hTRPM4 channel were obtained from the cryo-EM structure (PDB ID: 5WP6) [18] in the Protein Data Bank (https://www.rcsb.org/) [24]. Full-length TRPM4 has four identical subunits, which form an inverted crown-like structure. The latter includes the transmembrane domain (TMD), a large cytosolic domain formed by the N-terminal melastatin homology regions (MHR) and the C-terminal domain (CTD) (Fig 1A and 1B). The N-terminal part of each subunit has four melastatin homology regions (MHR1-4) and Pre-S1 helixes. TMD in each subunit contains six transmembrane segments (S1-S6). Segments S5 and S6, which are linked by a large extracellular membrane reentering P-loop, contribute a quarter to the pore module. In each subunit, segments S1-S4 form a voltage-sensing module (VSM). CTD contains TRP and CTD helices (Fig 1A and 1B). The MHR domain and the helical CTD constitute a unique intracellular architecture that distinguishes TRPM4 from any other TRP channel where the N-terminal cytosolic domains mainly contain ankyrin repeats [18].

### 2.4. Multiple sequence alignment and paralogue annotation

The paralogue annotation method identifies LP/P missense variants by transferring annotations across families of related proteins [13]. Earlier, we used a modified method of paralogue annotation to predict LP/P variants for the cardiac sodium channel hNav1.5 [12] and calcium channel hCav1.2 [11]. This approach is applied here to the hTRPM4 channel to select VUSs that are likely damaging variants.

For each paralogue channel, LP/P variants were collected as described in section 2.1. Amino acid sequences of hTRPM4 and paralogues channels were aligned using multiple sequence alignment program T-Coffee [25]. Proteins for which no LP/P variants were found were excluded from the alignment. Each paralogue variant was mapped on the hTRPM4 sequence (S2 Table) according to the alignment.

Disease-causing (LP/P) variants are more likely to occur at evolutionary conserved positions. Therefore we calculated the position-specific conservation score (Cs), which varies between 0 (no conservation), 0.8 (high conservation), and 1 (identical). Cs reflects the conservation of physico-chemical properties (small, polar, hydrophobic, tiny, charged, negative, positive, aromatic, aliphatic, proline) in the sequence alignment [26]. Cs values were calculated using the Zvelebil method [27] as implemented in the Amino Acid Conservation Calculation Service [28]. Variants in positions with conservation scores >0.3 were considered as LP/P variants according to [12, 13].

### 2.5. Sequence-based prediction of pathogenicity

Missense variants were annotated with 22 algorithm scores (REVEL, VEST4, MVP, CADD, LIST.S2, DANN, CenoCanyon, PrimateAI, DEOGEN2, M-CAP, MetaLR, MetaSVM, FATHMM, PROVEAN, Mutation Assessor, MutPred, PolyPhen2-HVAR, PolyPhen2-HDIV, SIFT, SIFT4G, LRT, Mutation Taster), which were obtained from database dbNSFPv4.2 [9]. To generate binary predictions (Damaging/Tolerated), we used the thresholds, which were determined as the optimal pathogenicity threshold from the AUC-ROC curve (Table 2).

The ‘*probably damaging*’ and ‘*possibly damaging*’ classes predicted by tool Polyphen were merged into a single ‘damaging’ class. The Mutation Assessor server subdivides mutants into four categories. Categories high (‘H’) or medium (‘M’) were treated as ‘Damaging’, whereas categories low (‘L’) or neutral (‘N’) were treated as ‘Tolerated’.

The overall prediction performance of the 22 methods was assessed by calculating sensitivity, specificity, Matthews Correlation Coefficient (MCC), and accuracy (ACC) as follows:

$$Sensitivity=\frac{TP}{TP+FN};$$

$$Specificity=\frac{TN}{TN+FP};$$

$$MCC=\frac{TP\times TN-FP\times FN}{\sqrt{\left(TP+FP)(TP+FN)(TN+FP)(TN+FN\right)}};$$

$$ACC=\frac{TP+TN}{TP+FP+TN+FN}.$$

The following abbreviations are used in these equations.

TP (true positive) is the number of disease-causing variants correctly predicted to be pathogenic;

FN (false negative) is the number of disease-causing variants incorrectly predicted as tolerated;

TN (true negative) is the number of neutral variants correctly predicted as tolerated;

FP (false positive) is the number of neutral variants incorrectly predicted as pathogenic;

MCC is a correlation coefficient between the observed and predicted binary classification, ranging from -1 (total disagreement between prediction and observation) to 1 (perfect prediction).

For the test dataset, we have chosen 103 benign and 130 LP/P variants from our broad dataset (Table 2, S1 Table). We also calculated the area under the ROC (Receiver Operating Characteristic) curve (AUC) using library pROC in programming language R. ROC curves were obtained by plotting sensitivity against (1 –specificity) at each threshold for each algorithm. The AUC can range from 0 (totally random) to 1 (perfectly correct prediction). We used AUC as the main measure of performance. The absence of a variant annotation negatively affects the accuracy of prediction. Thus, we have chosen only those algorithms, which predicted pathogenicity for over 30% variants in our dataset (S1 Table).

## 3. Results

### 3.1. Assessment of missense variants in hTRPM4 and its paralogues

Overall, for TRP channels listed in Table 1, we collected 103 benign variants (with AF > 0.00005), 130 LP/P variants,and 1,117 VUSs (Table 2, S1 Table). The largest numbers of LP/P variants were found for channels hTRPV4, hTRPM1 and hTRPC6 (72, 23, and 20, respectively). No LP/P variants were found for channels hTRPC1, hTRPC4, hTRPC5, hTRPC7, hTRPM2, hTRPM3, hTRPM5, hTRPM7, hTRPM8, hTRPV1, hTRPV2, and hTRPV5 as well for channel families TRPP and hTRPML. For channel hTRPM4, we found 10 LP/P variants, 6 benign variants and 565 VUSs (Table 2 and S1 Table).

### 3.2. Distribution of pathogenic variants in topological regions of hTRPM4

Most of pathogenic variants are localized in the cytoplasmic part of the channel, mainly in alpha-helices of the MHR regions. Two variants, I1033M and I1040T, were found in segment S6, and one variant (Y790H) in segment S1 (Fig 1A and 1B). Most of LP/P variants of hTRPM4 (83%) are associated with the PFHBI syndrome. Other variants are associated with erythrokeratodermia and Brugada syndrome (S1 Table).

### 3.3. Comparison of *in silico* bioinformatics tools

**Fig 2:**
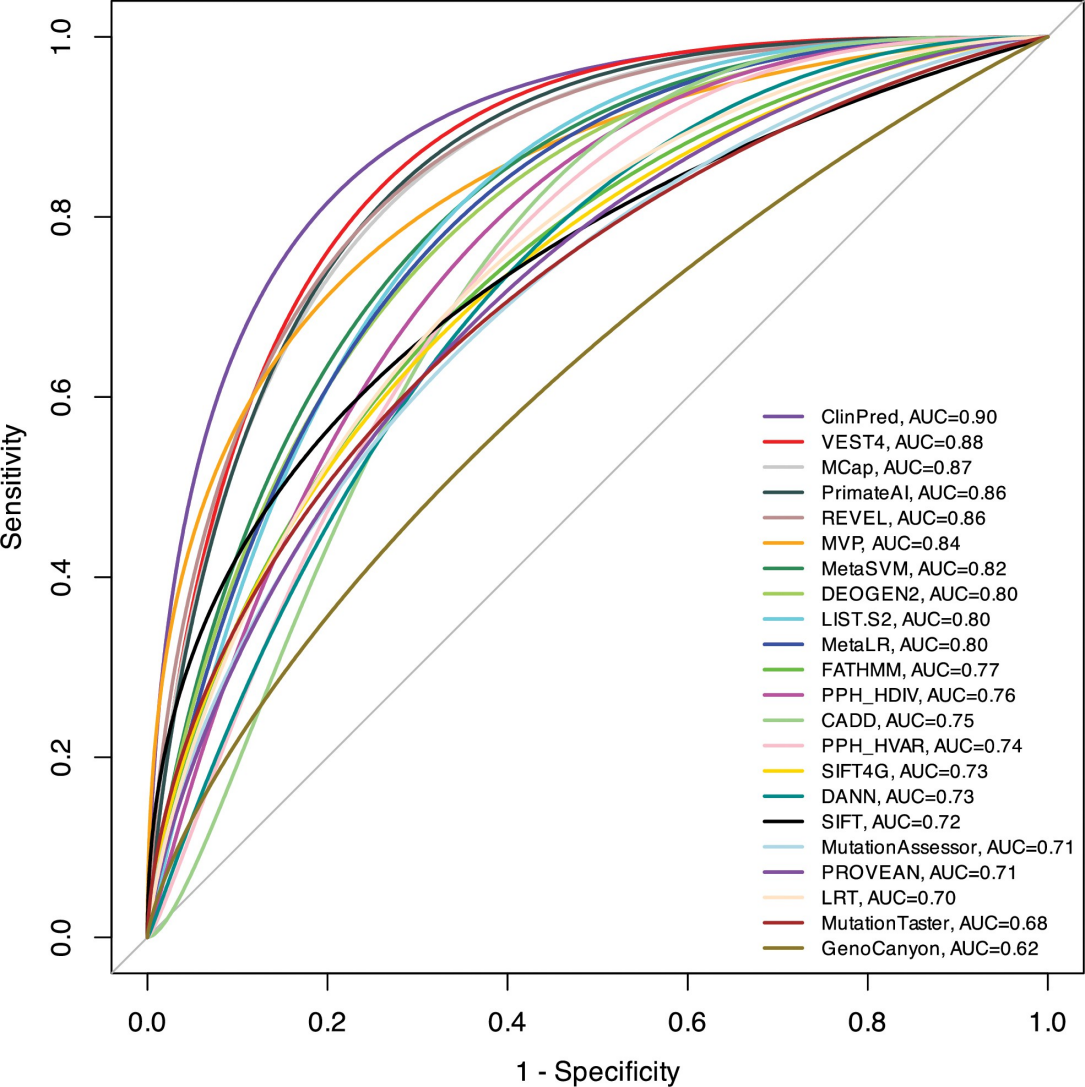
ROC curves for prediction algorithms on the broad dataset. This plot illustrates performance of quantitative predictions. The higher AUC score indicates the better performance.

We compared performance of 22 variant interpretation tools (Table 3, Fig 2). We first compiled a test set with 130 true positive (TP) observations and 103 true negative (TN) observations obtained from our broad dataset (S1 Table). AUC and ROC curves are shown in Fig 2. For each tool, we found an optimal deleterious threshold for binary predictions based on its ROC curve, which shows the sensitivity and specificity corresponding to different score thresholds (Table 3).

**Table 3:** Performance of variant interpretation tools.

Tool	Deleterious threshold	Sensitivity	Specificity	MCC	ACC	AUC
ClinPred	>0.6	0.92	0.75	0.68	0.83	0.90
VEST4	>0.65	0.87	0.72	0.59	0.79	0.88
MCap	>0.05	0.91	0.6	0.54	0.76	0.87
REVEL	>0.45	0.84	0.72	0.56	0.78	0.86
PrimateAI	>0.6	0.92	0.69	0.62	0.8	0.86
MVP	>0.7	0.86	0.62	0.5	0.74	0.84
MetaSVM	>0	0.62	0.78	0.4	0.7	0.82
MetaLR	>0.4	0.72	0.7	0.42	0.71	0.8
LIST.S2	>0.85	0.9	0.56	0.49	0.73	0.8
DEOGEN2	>0.4	0.8	0.61	0.42	0.71	0.8
FATHMM	<-1	0.63	0.76	0.39	0.69	0.77
PPH_HDIV	>0.45	0.9	0.45	0.4	0.68	0.76
CADD	>3	0.83	0.58	0.43	0.71	0.75
PPH_HVAR	>0.45	0.85	0.57	0.44	0.71	0.74
SIFT4G	<0.05	0.76	0.62	0.39	0.69	0.73
DANN	>0.99	0.88	0.46	0.38	0.67	0.72
SIFT	<0.0045	0.63	0.71	0.34	0.67	0.72
PROVEAN	<-1.5	0.81	0.43	0.26	0.62	0.71
Mutation Assessor	>1.7	0.79	0.51	0.31	0.65	0.71
LRT	<1e-04	0.88	0.49	0.41	0.7	0.7
Mutation Taster	>1	0.69	0.64	0.33	0.67	0.68
GenoCanyon	>0.7	0.78	0.35	0.14	0.56	0.62

Deleterious threshold is the custom pathogenicity threshold that divides variants in two categories: pathogenic or benign. The larger or smaller the score than the threshold for specific tool, the more likely the variant is damaging. Sensitivity characterizes the number of LP/P variants, which were predicted as LP/P by the tool, while specificity characterizes the number of benign variants, which were predicted as benign by the tool. Accuracy indicates the predictive accuracy of the tool.

ClinPred demonstrated the best performance in predicting pathogenicity for variants in the TRP superfamily (accuracy = 0.83, AUC = 0.90) (Fig 2), followed by VEST4 (accuracy = 0.79, AUC = 0.88) and MCap (accuracy = 0.76, AUC = 0.87). With the score threshold of 0.6 ClinPred has a relatively high tendency of correctly classifying benign variants as tolerated (specificity = 0.75) and LP/P variants as pathogenic (sensitivity = 0.92) (Table 3). REVEL, PrimateAI, MVP, MetaSVM, MetaLR, LIST.S2 and Deogen2 performed with AUC of > 0.80. The AUC of other algorithms ranged from 0.70 to 0.80. The lowest accuracy across all methods was found for GenoCanyon (accuracy = 0.56, AUC = 0.62).

### 3.4. Paralogue annotation of variants identified in hTRPM4

We mapped the LP/P variants of paralogs onto the hTRPM4 regions basing on the multiple sequence alignments (section 2.4, S2 Table). A total of 63 known LP/P variants in paralogues are mapped to 36 amino acid positions in the hTRPM4 channel (S2 Table). In these positions, we found 51 variants of hTRPM4, including 50 VUSs and one benign variant. In some cases, more than one variant was mapped into one sequence position. Twenty LP/P variants in paralogues TRP channels were mapped in the MHR1/2 region of hTRPM4 (S2 Table). The MHR1/2 region is ~ 30% of the entire sequence and it is highly variable among paralogues. Thus, highly conserved regions among paralogues are transmembrane segments S1-S6 and the TRP helix. These regions have more than 60% of conserved positions (Cs>0.3).

### 3.5. Consensus prediction LP/P variants in hTRPM4 by ClinPred and paralogs annotation method

**Fig 3:**
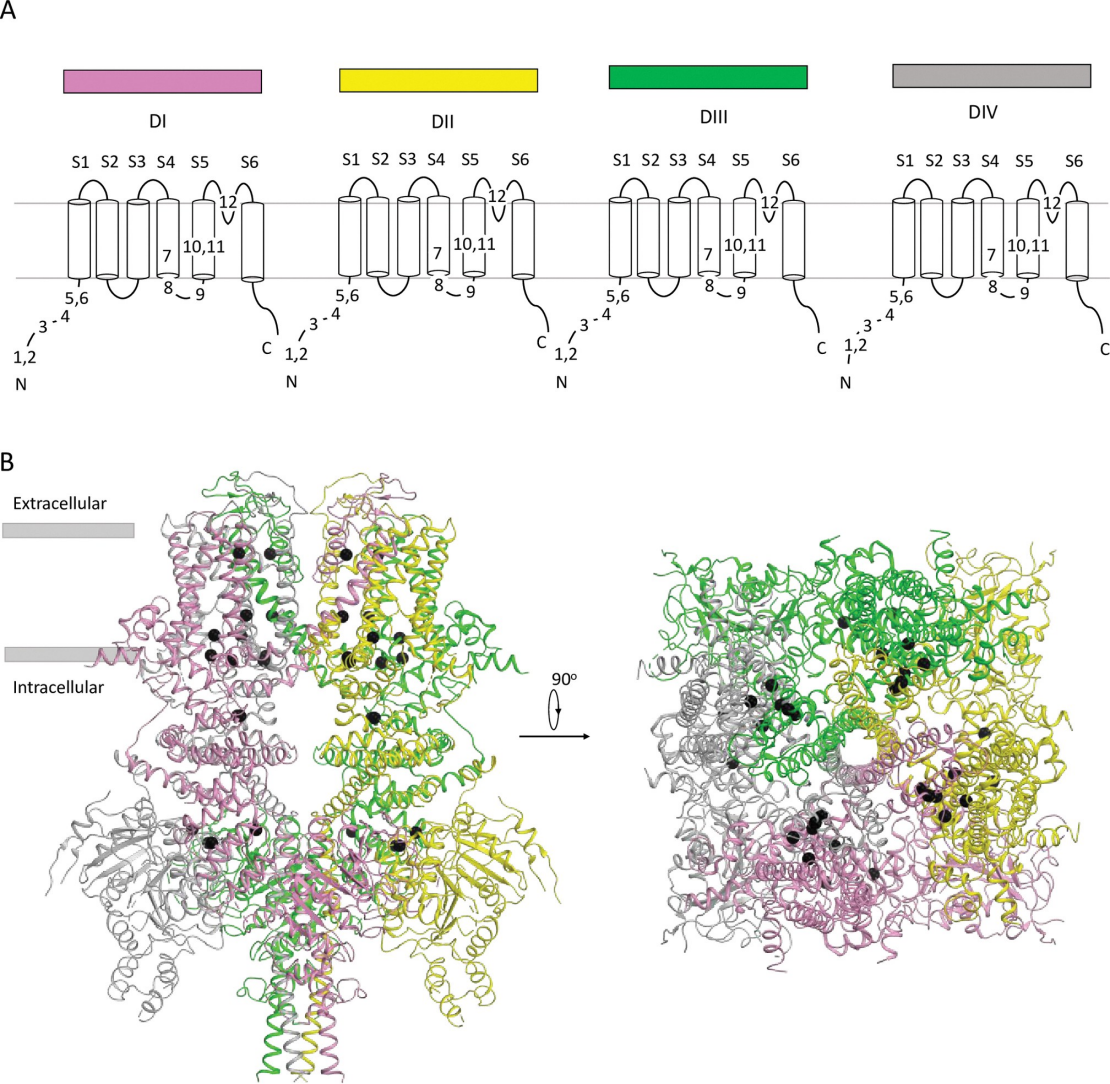
Channel hTRPM4. (A) Topology of transmembrane helices. VUSs with high ClinPred and paralogue annotation scores are indicated by their numbers in Table 4. The number of variants in the full-fledged channel is four times larger than that in a subunit. (B) Side and intracellular views of the cryo-EM structure [18]. Subunits are colored as top bars in (A). Positions of hTRPM4 VUSs are shown as spheres.

Most of hTRPM4 variants in our dataset are currently classified as VUS. ClinPred predicted 299 VUSs with the pathogenicity score > 0.6 as LP/P variants. Among these, we further selected those variants, which are annotated as LP/P in at least one paralog of hTRPM4 (conservation score across paralogues Cs>0.3). Both methods consensually predicted 12 of 565 VUSs as LP/P variants (Table 4 and Fig 1). The variants are localized mainly in the transmembrane region of the channel (Fig 1). Five variants are located in transmembrane helices S4, S5 and one variant (V966I) is located in the P-loop between helices S5 and S6. Fig 3 indicates parental residues of the 12 variants in the cryo-EM structure of hTRPM4 [18]. In the homo-tetrameric channel, each parental residue is shown four times.

**Table 4:** hTRPM4 VUS variants with high ClinPred and paralogue annotation scores.

#	hTRPM4 Variant	Secondary structure	Location	Paralogue	
				Channel	Variant
1	M1V	Disordered	N-end	TRPM1	M1I
2	M1T	Disordered	N-end	TRPM1	M1I
3	R437W	α-helix	MHR3	TRPM1	R473P
4	I445M	α-helix	MHR3	TRPV6	I223T
5	R664H	α-helix	MHR4	TRPM1	R721Q
6	R664L	α-helix	MHR4	TRPM1	R721Q
7	I909V	α-helix	S4	TRPV4	F592L
8	N913S	Disordered	S4-S5	TRPV4	L596P
9	G917R	Disordered	S4-S5	TRPV4	G600E
				TRPV3	G573C/S
10	F936I	Alpha-helix	S5	TRPV4	V620I
11	F936L	Alpha-helix	S5	TRPV4	V620I
12	V966I	Alpha-helix	P-loop	TRPM1	I1002F

### 3.6. Intersegment contacts between parental residues of ClinPred predicted LP/P variants

We used the cryo-EM structure of the hTRPM4 channel to find intersegment contacts between the parental (WT) residues for which ClinPred predicted a high probability of pathogenicity of respective VUSs (S3 Table). We reasoned that if two sidechains of such residues have heavy atoms with 5 Å from each other, then mutation of any contact partner would affect the intersegment interaction and relative stability of the channel state and thus may underlie the channel dysfunction.

**Fig 4:**
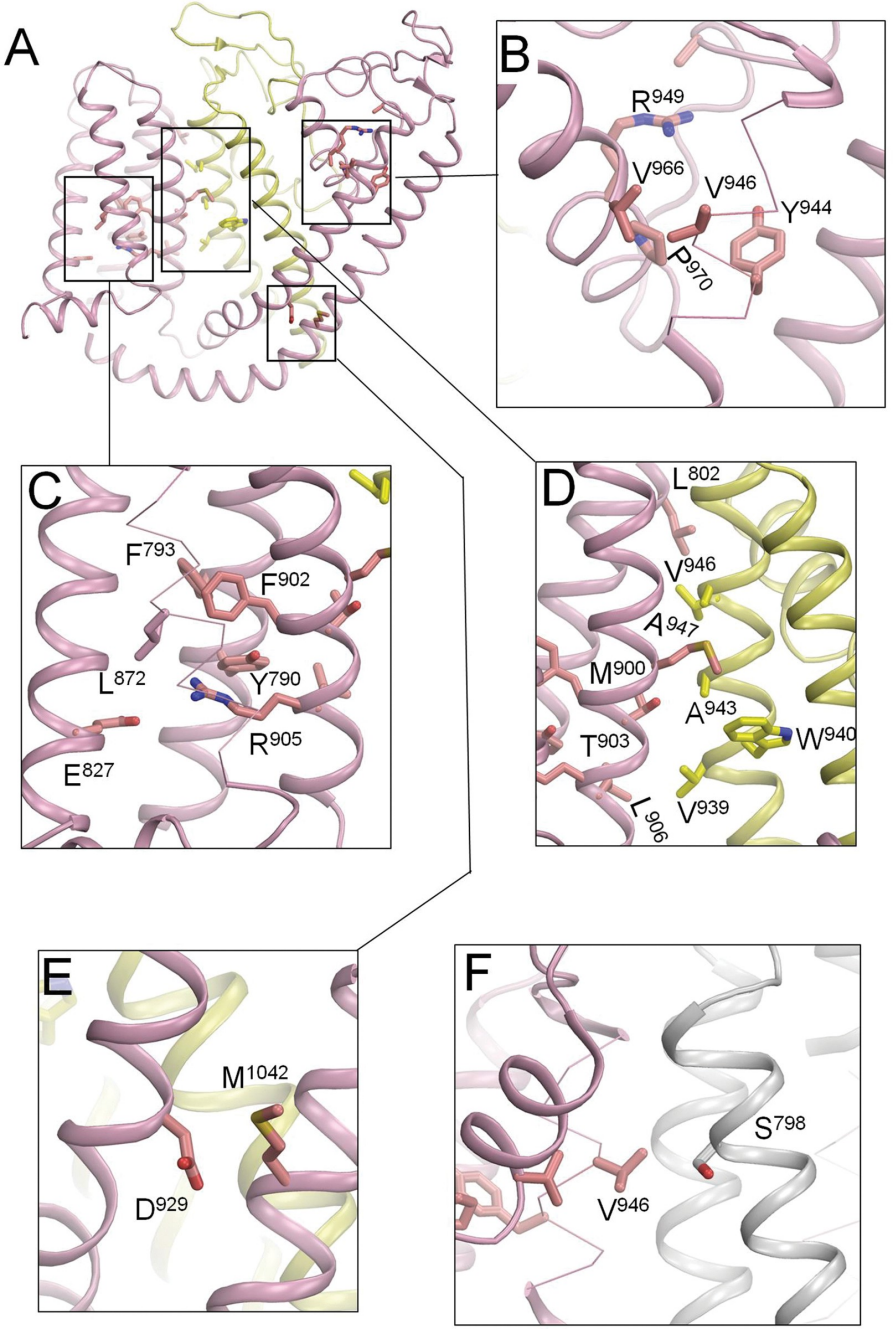
Subunit interface in transmembrane and extracellular region in the cryo-EM structure of hTRPM4. Shown are intersegment contacts between parental (WT) residues of ClinVar listed VUSs that according to ClinPred have a high probability to be damaging variants. See Table 5 for the list of contacts.

In the transmembrane and extracellular parts of the channel, we found 16 contacts between the parental residues and propose that respective 25 VUSs have a high probability to be LP/P variants (Table 5 and Fig 4). Most of the found contacts are within the VSM, which undergoes significant transformation in the cryo-EM structures of P-loop channels with activated and deactivated VSMs [29, 30]. The cryo-EM structure of hTRPM4 shows activated VSMs. Although the voltage dependency of the channel is rather weak [31], such contacts will change upon the VSM deactivation. Mutations in such contacts (annotated “*VSM activation*” in Table 5) would affect VSM activation/deactivation. Contacts between the sliding helix S4 and the outer helix S5 may mediate signal transmission from VSM to the pore module [32]. Mutations of such contacts may cause the channel dysfunction by affecting the signal transmission. Respective contacts are annotated “*VSM*_*i*_*-PM*_*i+1*_ *signal*” in Table 5.

**Table 5:** Intersegment contacts in the cryo-EM structure between WT residues in the transmembrane and P-loop regions with high ClinPred score ^a^.

Contact #^b^	Variant #	VUS		Contact		Contact	Likely impact of mutation
				VUS		Change upon	
1	1	Y790C^c^	S1_I_	R905W	S4_I_	S4 sliding	VSM Activation
2	2	F793C	S1_I_	R905W	S4_I_	S4 sliding	VSM Activation
3	3	S798L	S1_IV_	V946A	S5_I_		VSM_i_-PM_i+1_ Clamp
4	4	L802F	S1_I_	V946A	S5_II_		VSM_i_-PM_i+1_ Clamp
5	5	E827K	S2_I_	R905W	S4_I_	S4 sliding	VSM Activation
6	6	L872V	S3_I_	F902L	S4_I_	S4 sliding	VSM Activation
7	7	M900I	S4_I_	W940C	S5_II_	S4 sliding	VSM_i_-PM_i+1_ signal
6’	8	F902L	S4_I_	L872V	S3_I_	S4 sliding	VSM Activation
8	9	T903M	S4_I_	A943D	S5_II_	S4 sliding	VSM_i_-PM_i+1_ signal
2’	10	R905W	S4_I_	F793C	S1_I_	S4 sliding	VSM Activation
1’		"	S4_I_	Y790C	S1_I_	S4 sliding	VSM Activation
9	11	L906P	S4_I_	V939L	S5_II_	S4 sliding	VSM-PM signal
10	12	V923L	S4-S5 _I_	F936L_II_	S5_II_	S4 sliding	VSM-PM signal
11	13	D929E	S5_I_	M1042I	S6_I_		Activation gating
12	14	V939L	S5_II_	T903M	S4_I_	S4 sliding	VSM_i_-PM_i+1_ signal
9’		"	S5_II_	L906P	S4_I_	S4 sliding	VSM_i_-PM_i+1_ signal
7’	15	W940C	S5_II_	M900I	S4_I_	S4 sliding	VSM_i_-PM_i+1_ signal
8’	16	A943D	S5_II_	T903M	S4_I_	S4 sliding	VSM_i_-PM_i+1_ signal
13	17	Y944C	S5_I_	P970S	P1_I_		PM_i_ folding
14	18	V946A	S5_I_	V966I	P1_I_		PM_i_ folding
3’		"	S5_I_	S798L	S1_IV_		VSM_i_-PM_i+1_ Clamp
4’		"	S5_II_	L802F	S1_I_		VSM_i_-PM_i+1_ Clamp
15	19	A947D	S5_II_	M900I	S4_I_	S4 sliding	VSM_i_-PM_i+1_ signal
14’	20	V966I	P1_I_	V946A	S5_I_		PM_i_ folding
16	21	R969L	P1_I_	D982N	P2_I_		P-loop folding
16’	22	R969H	P1_I_	D982N	P2_I_		P-loop folding
13’	23	P970S	P1_I_	Y944C	S5_I_		PM_i_ folding
16’	24	D982N	P2_I_	R969L	P1_I_		P-loop folding
16’		"	P2_I_	R969H	P1_I_		P-loop folding
11’	25	M1042I	TRP_I_	D929E	S5_I_		

^a^ The following variant conditions are reported in ClinVar: Progressive familial heart block type IB (15 variants), Cardiovascular Phenotype (7 variants), or Not Provided (2 variants)

^b^ Each contact is indicated twice to show both contacts partners.

^c^ Gain-of-expression and gain of-function [52].

We also found intersegment contacts whose state-dependency is unclear (between P-loop helices P1 and P2) or unlikely (between helices S5 and P1). In practically all P-loop channels, the C-terminal part of the outer helices (S5) has a small residue (G, A, or S), which is involved in a knob-into-hole contact with a bulky residue at the N-end of P-helix of the same subunit/repeat [33]. Mutations in such contacts, which would affect folding of the respective subunit/repeat in the pore module, are annotated “*PM*_*i*_ *folding*” in Table 5.

Four intra-subunit contacts between P-loop helices P1 and P2 are involved in stabilization of the P-loop folding (annotated “*P-loop folding*” in Table 5). In the Nav1.5 channel, disease mutations in such contacts affect slow inactivation [34]. Structural determinants of TRPM4 inactivation are unclear, but in the TRPM2 channel inactivation gate is located in the extracellular selectivity filter [35].

We also found four inter-subunit contacts at the extracellular part of the channel between residues in the VSM helix S1 and PM helix S5. The state-dependency of such contacts is unclear, but respective interactions likely contribute to stabilizing mutual disposition of VSMs and PM.These contacts are annotated “*VSM*_*i*_*-PM*_*i+1*_ *Clamp*” in Table 5.

**Fig 5:**
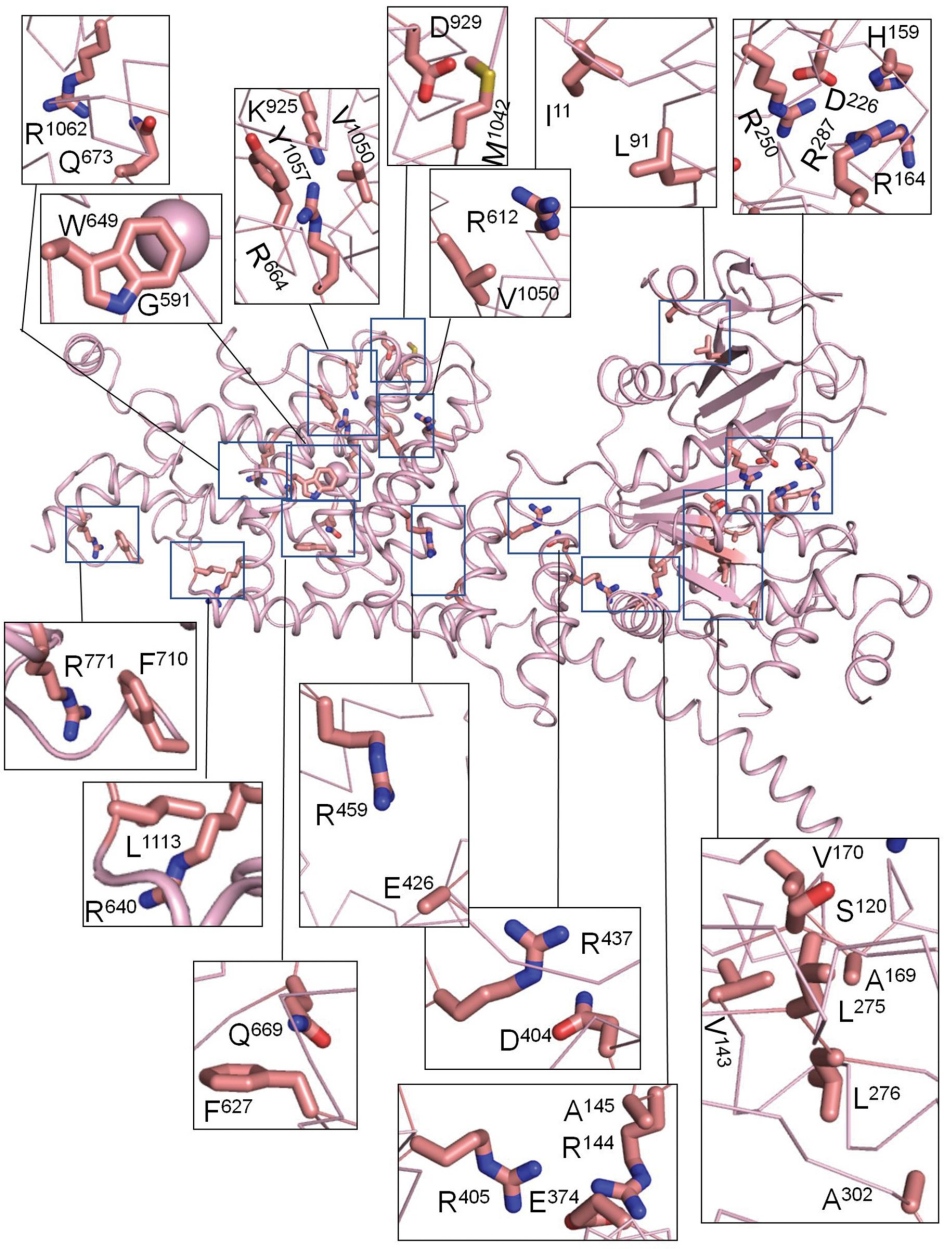
Cytoplasmic part in the cryo-EM structure of hTRPM4. Shown are intersegment contacts between parental residues of ClinVar reported VUSs that according to ClinPred have a high probability to be LP/P variants. See Table 6 for the list of contacts.

We further found 24 intersegment contacts in the cytoplasmic parts of the channel between parental residues of VUSs with high ClinPred score of pathogenicity. These contacts involve 43 residues (Table 6 and Fig 5). In lack cryo-EM structures with different states of the cytoplasmic parts, which are unique in TRP channels, state-dependency of such contacts is unclear. Nevertheless, the fact that some experimentally confirmed pathogenic mutations are located in this region (Fig 1) suggests functional importance of such contacts. For example, LP/P variants in domain MHR1/2 may disturb the channel sensing of external stimuli [18].

**Table 6:** Cytoplasmic segments of hTRPM4 in the cryo-EM structure: Contacts between side chains of parent residues of VUSs’ with high ClinPred score ^a^.

#	VUS		Contact		#	VUS		Contact	
1	I11V	MHR1/2	L91F	MHR3	21	N404K	MHR3	R437W	MHR3
2	L91F	MHR3	I11V	MHR1/2	22	R405P	MHR3	E374Q	MHR1/2
3	S120L	MHR1/2	R250H	MHR1/2		“	MHR3	E374K	MHR1/2
	“	MHR1/2	L275F	MHR1/2	23	E426K	MHR3	R459L	MHR3
4	V143L	MHR1/2	A169T	MHR1/2	24	R437W	MHR3	N404K	MHR3
	“	MHR1/2	V170I	MHR1/2	25	R459L	MHR3	E426K	MHR3
5	R144Q	MHR1/2	E374Q	MHR1/2	26	G591R	MHR4	W649L	MHR4
6	R144W	MHR1/2	E374K	MHR1/2	27	L596P	MHR4	R612K	MHR4
7	A145V	MHR1/2	E374K	MHR1/2	28	R612K	MHR4	L596P	MHR4
8	R250H	MHR1/2	S120L	MHR1/2	29	F627S	MHR4	Q669R	MHR4
9	H159Q	MHR1/2	R164W	MHR1/2	30	R640C	MHR4	L1113V	TRP-CTD
10	R164W^b^	MHR1/2	H159Q	MHR1/2	31	W649L	MHR4	G591R	MHR4
11	A169T	MHR1/2	V143L	MHR1/2	32	R664L	MHR4	Y1057C	TRP_helix
12	V170I	MHR1/2	V143L	MHR1/2	33	Q669R	MHR4	F627S	MHR4
13	D226E	MHR1/2	H159Q	MHR1/2	34	Q673E	MHR4	R1062G	TRP_helix
14	R250H	MHR1/2	R287L	MHR1/2	35	F710C	MHR4	R771C	MHR4
15	L275F	MHR1/2	S120L	MHR1/2	36	R771C	MHR4	F710C	MHR4
16	L276P	MHR1/2	A302V	MHR1/2	37	K925T	S4-S5	V1050A	TRP_helix
17	R287L	MHR1/2	R250H	MHR1/2	38	D929	S5	M1042	TRP_helix
18	A302V	MHR1/2	L276P	MHR1/2	39	M1042	TRP_helix	D929	S5
19	E374Q	MHR1/2	R405P	MHR3	40	V1050A	TRP_helix	K925T	S4-S5
20	E374K	MHR1/2	R405P	MHR3	41	Y1057C	TRP_helix	R664L	MHR4
	“	MHR1/2	A145V	MHR1/2	42	R1062G	TRP_helix	Q673E	MHR4
	“	MHR1/2	R144W	MHR1/2	43	L1113V^c^	TRP-CTD	R640C	MHR4

^a^ All contacts are within single subunit; No intersubunit contact were found

^b^ GoF [53]

^c^ Gain-of-expression and gain of-function [52]

## 4. Discussion

Over 90% of variants in the hTRPM4 channel are classified as VUSs.The large proportion of VUSs is a serious problem because patients with respective mutations are not clinically actionable and incorrect interpretation of the pathogenicity of the variant has serious ramifications for assessing a probability of sudden cardiac death [36–38]. The ACMG/AMP guidelines suggest using *in silico* prediction tools for variant interpretation. Due to their low accuracy, computational approaches are still considered as rather weak supporting evidence compared to functional methods. Some of the early popular variant interpretation tools like SIFT are relatively simple and rely on sequence homology and physico-chemical properties of amino acids [39]. Other earlier tools use one or more structure-based characteristics such as effects of mutations on the stability, folding and dynamics of proteins, e.g. I-Mutant 2.0 [40], FoldX [41], and CUPSAT [42] or combine sequence and structure characteristics, e.g. PolyPhen-2 [43]. Most of these tools use machine learning methods trained with numerous biochemical features and evolutionary constraints or pathogenic classification data that can be collected from structure-based databases, e.g. the thermodynamic database for proteins and mutants [44] and variant pathogenicity databases such as SWISS-PROT [20] and ClinVar [7]. Recently developed tools like REVEL, MetaSVM, and MCap employ multiple scores from different tools into a single ensemble score with improved prediction capabilities [45]. ClinPred, besides a wide range of existing approaches, uses allele frequency from gnomAD as one of the key predictive features [46].

Our recent analysis revealed that various popular bioinformatics tools yield different predictions of pathogenicity for known LP/P and benign variants of the hCav1.2 channel and its paralogues [11]. We have shown that for each protein family it is necessary to select a specific best-performing predictor from a variety of existing methods. This is possible if databases describe rather large number of pathogenic and neutral variants for the given family of proteins. In the present study, we have shown that for the TRP superfamily, ClinPred is the most accurate method with accuracy of 0.83 and AUC of 0.90.

We further used the paralogue annotation method, which employs multiple sequence alignment and data on missense variants associated with diseases across the protein family. This approach was developed and experimentally validated with a large set of known variants in eight genes associated with the long QT syndrome, and demonstrated positive predictive value of 98.4% in these genes [47]. The accuracy of the method depends on the quality of the protein sequence alignment, the conservation score Cs in each sequence position among paralogues, and the quality of the evidence relating genotype to phenotype for the paralogue variant [13]. The lack of paralogue annotation for an uncharacterized variant in the protein in question does not mean the variant is non-pathogenic. In other words, the paralogue annotation method is based on clinical genotype–phenotype relationships in humans, rather than on computational prediction. However, when the paralogue annotation method is applied along with a bioinformatics tool, the consensus predictions of pathogenicity become much more reliable than predictions from individual methods.

The hTRPM4 channel belongs to the transient receptor potential (TRP) superfamily. The TRPM family, which has eight members, is the largest and most diverse subfamily of the TRP channels [48]. Since very few LP/P variants of the TRPM channels were found in public databases, we also considered data on variants from other TRP family members (Table 1). However, only TRPA1, TRPC6, TRPV4, and TRPV6 channels, for which one or more LP/P variantsare described, were included to our big dataset (Table 2, S1 Table).

All hTRPM4 paralogues are homotetramers with conserved TMD and TRP domains. TRPM channels also have a MHR domain in the N-terminal region, which distinguishes them from other TRP channels where the N-terminal cytosolic domains contain mainly ankyrin repeats. Therefore, TRPM4 residues in the MHR region have low conservation scores (S2 Table). The MHR domain is subdivided into four melastatin homology regions (MHR1-MHR4) based on sequence similarity within the TRPM family [49]. Most of known LP/P variants are localized in MHR1/2 domain (Fig 1) and are associated with the PFHBI-IB disease [48]. Domain MHR1/2 consists of a β-sheet core surrounded by α helices and loops (Fig 1). It strongly interacts with domain MHR3 from the same subunit, and has rather weak contacts with domain MHR3 in the adjacent subunit. The interface between domains MHR1/2 and MHR3 forms a binding pocket for decavanadate, a negatively charged metal cluster that shifts the voltage dependent activation of the hTRPM4 channel towards negative potentials [18, 50]. It was proposed that LP/P variants in domain MHR1/2 may disturb sensing of external stimuli by the channel [18].

Using ClinPred and paralogue annotations, we predicted that 12 VUSs of the hTRM4 are LP/P variants. All these variants are located in the MHR and TMD regions. Two variants are at residue positions 437 and 445 in the α14 helix of MHR3 (Fig 1). In the cryo-EM structure of hTRPM4 [18], helices α14 and α13 form an interface, and variants R437W and I445M may affect the helices movement and destabilize the MHR3 domain [51].The stacked α-helices of MHR3-4 form interfaces with the C-terminal TRP domain, thus linking the N- and C-termini and additionally providing a direct interaction between the cytosolic regions of the channel and the transmembrane core. Variants R664H and R664L are located in domain MHR4 that interacts with the TRP domain and the S2–S3 linker helix of TMD on the top, and with the rib helix of CTD and MHR3 on the bottom. Mutated residues N913S and G917R in the S4–S5 linker form hydrogen bonds with residues in the TRP domain [18]. Variants R664H, R664L, N913S, and G917R, which we characterized as LP/P, may affect interactions between MHR, CTD, and TMD. Three variants, F936I, F936L and V966L, were found in the S5 segment and P-loop region, which belongs to the pore domain. These variants may influence gating of TRPM4 and calcium permeability.

Among 10 pathogenic variants reported in public databases, only two missense variants are located in the TM region: K914R andI1033M. Side chain of K914 is unresolved in the cryo-EM structure. I1033 makes multiple intersubunit contacts with hydrophobic residues (F933 in P1, F975 in S5, L1039 and L1043 in S6), but none of these residues is reported in public databases or has a high score by ClinPred, suggesting that I1033M may affect channel expression rather than the channel function.

Among six VUSs located in the transmembrane region that ClinPred and paralogues annotation method consensually predicted as LP/P variants (I909V, N913S, G917R, F936I, F936L and V966I), only parental residue I909 forms intersegment contact with a residue whose pathogenic variant Y790C is reported in public databases. Y790 forms an intersegment contact with R905, the only basic residue in the voltage-sensing sliding helix S4 (Table 5). A recent study identified TRPM4 variant Y790C in patients suffering from complete heart block and their relatives and demonstrated gain-of-expression and gain-of-function of mutant channel Y790C [52]. The same study also confirmed pathogenic status of variant L1113V and demonstrated gain-of-expression and gain-of-function of mutant channel L1113V. These findings exemplify predicting potential of our approaches.

## 5. Conclusions

In this study we compiled and analyzed a broad dataset that includes all currently known pathogenic and likely pathogenic variants in the hTRPM4 channel and its seven paralogues. We found that ClinPred is the best-performing bioinformatics tool to predict likely pathogenic/pathogenic (LP/P) variants for TRP channels. ClinPred and the paralogue annotation method consensually predicted 12 hTRPM4 VUSs as potentially pathogenic variants. We further used a cryo-EM structure of the hTRPM4 channel to analyze intersegment contacts of 307 parental (wild-type) residues, which have a high ClinPred score of pathogenicity. We found scores of contact pairs between the WT residues whose mutations may affect the protein structure. Taken together, our approaches predicted that a total of 80 VUSs are likely damaging variants. The latter number is much larger than 10 pathogenic variants currently reported in Humsavar, ClinVar and Ensembl Variation. The 80 variants are promising targets for further experimental and theoretical studies.

## Supporting information

S1 Table Broad dataset of hTRPM4 variants and its paralogues. (XLSX)

S2 Table Mapping of LP/P paralogue variants on human TRPM4 sequence. (XLSX)

S3 Table ClinPred predicted VUS variants with the score > 0.6. (XLSX)

## Abbreviations

ACMG/AMP: The American College of Medical Genetics and Genomics and the Association for Molecular Pathology
AF: Allele Frequencies
AUC: Area Under the Curve
AVB: Atrioventricular conduction Block
CIP: Conflicting Interpretation of Pathogenicity
Cs: position-specific Conservation Score
CTD: C-Terminal Domain
ICCD: Isolated Cardiac Conduction Disease
MCC: Matthews Correlation Coefficient
MHR: Melastatin Homology Regions
LP/P: Likely Pathogenic/Pathogenic variant
PFHBI: Progressive Familial Heart Block type I
PM: Pore module
ROC: Receiver Operator Curve
S1-S6: transmembrane segments
TMD: Transmembrane Domain
VSM: Voltage-Sensing Module
VUS: Variant of Uncertain clinical Significance

## References

[1] Daumy X, Amarouch MY, Lindenbaum P, Bonnaud S, Charpentier E, et al. (2016) Targeted resequencing identifies TRPM4 as a major gene predisposing to progressive familial heart block type I. Int J Cardiol 207: 349–358. doi: 10.1016/j.ijcard.2016.01.052 26820365

[2] Syam N, Chatel S, Ozhathil LC, Sottas V, Rougier JS, et al. (2016) Variants of Transient Receptor Potential Melastatin Member 4 in Childhood Atrioventricular Block. J Am Heart Assoc 5. doi: 10.1161/JAHA.114.001625 27207958 PMC4889160

[3] Demion M, Thireau J, Gueffier M, Finan A, Khoueiry Z, et al. (2014) Trpm4 gene invalidation leads to cardiac hypertrophy and electrophysiological alterations. PLoS One 9: e115256. doi: 10.1371/journal.pone.0115256 25531103 PMC4274076

[4] Liu H, Chatel S, Simard C, Syam N, Salle L, et al. (2013) Molecular genetics and functional anomalies in a series of 248 Brugada cases with 11 mutations in the TRPM4 channel. PLoS One 8: e54131. doi: 10.1371/journal.pone.0054131 23382873 PMC3559649

[5] Jacobs G, Oosterlinck W, Dresselaers T, Geenens R, Kerselaers S, et al. (2015) Enhanced beta-adrenergic cardiac reserve in Trpm4(-)/(-) mice with ischaemic heart failure. Cardiovasc Res 105: 330–339. 25600961 10.1093/cvr/cvv009

[6] Earley S (2013) TRPM4 channels in smooth muscle function. Pflugers Arch 465: 1223–1231. doi: 10.1007/s00424-013-1250-z 23443854 PMC3686874

[7] Landrum MJ, Lee JM, Benson M, Brown G, Chao C, et al. (2016) ClinVar: Public archive of interpretations of clinically relevant variants. Nucleic Acids Research 44: D862–D868. doi: 10.1093/nar/gkv1222 26582918 PMC4702865

[8] Ghosh R, Oak N, Plon SE (2017) Evaluation of in silico algorithms for use with ACMG/AMP clinical variant interpretation guidelines. Genome Biology 18: 1–12. 29179779 10.1186/s13059-017-1353-5 PMC5704597

[9] Dong C, Wei P, Jian X, Gibbs R, Boerwinkle E, et al. (2015) Comparison and integration of deleteriousness prediction methods for nonsynonymous SNVs in whole exome sequencing studies. Hum Mol Genet 24: 2125–2137. doi: 10.1093/hmg/ddu733 25552646 PMC4375422

[10] Anderson D, Lassmann T (2018) A phenotype centric benchmark of variant prioritisation tools. npj Genomic Medicine 3: 5. doi: 10.1038/s41525-018-0044-9 29423277 PMC5799157

[11] Tarnovskaya SI, Kostareva AA, Zhorov BS (2021) L-Type Calcium Channel: Predicting Pathogenic/Likely Pathogenic Status for Variants of Uncertain Clinical Significance. Membranes (Basel) 11. doi: 10.3390/membranes11080599 34436362 PMC8399957

[12] Tarnovskaya SI, Korkosh VS, Zhorov BS, Frishman D (2020) Predicting novel disease mutations in the cardiac sodium channel. Biochem Biophys Res Commun 521: 603–611. doi: 10.1016/j.bbrc.2019.10.142 31677787

[13] Walsh R, Peters NS, Cook SA, Ware JS (2014) Paralogue annotation identifies novel pathogenic variants in patients with Brugada syndrome and catecholaminergic polymorphic ventricular tachycardia. Journal of medical genetics 51: 35–44. doi: 10.1136/jmedgenet-2013-101917 24136861 PMC3888601

[14] Sun Y, Sukumaran P, Schaar A, Singh BB (2015) TRPM7 and its role in neurodegenerative diseases. Channels (Austin) 9: 253–261. doi: 10.1080/19336950.2015.1075675 26218331 PMC4826135

[15] Kruse M, Pongs O (2014) TRPM4 channels in the cardiovascular system. Curr Opin Pharmacol 15: 68–73. doi: 10.1016/j.coph.2013.12.003 24721656

[16] Zierler S, Hampe S, Nadolni W (2017) TRPM channels as potential therapeutic targets against pro-inflammatory diseases. Cell Calcium 67: 105–115. doi: 10.1016/j.ceca.2017.05.002 28549569

[17] Vennekens R, Mesuere M, Philippaert K (2018) TRPM5 in the battle against diabetes and obesity. Acta Physiol (Oxf) 222. doi: 10.1111/apha.12949 28834354

[18] Winkler PA, Huang Y, Sun W, Du J, Lu W (2017) Electron cryo-microscopy structure of a human TRPM4 channel. Nature 552: 200–204. doi: 10.1038/nature24674 29211723

[19] UniProt C (2015) UniProt: a hub for protein information. Nucleic Acids Res 43: D204–212. doi: 10.1093/nar/gku989 25348405 PMC4384041

[20] Boutet E, Lieberherr D, Tognolli M, Schneider M, Bansal P, et al. (2016) UniProtKB/Swiss-Prot, the Manually Annotated Section of the UniProt KnowledgeBase: How to Use the Entry View. Methods in molecular biology (Clifton, NJ) 1374: 23–54. doi: 10.1007/978-1-4939-3167-5_2 26519399

[21] Karczewski KJ, Francioli LC, Tiao G, Cummings BB, Alföldi J, et al. (2020) The mutational constraint spectrum quantified from variation in 141,456 humans. Nature 581: 434–443. doi: 10.1038/s41586-020-2308-7 32461654 PMC7334197

[22] Kaltman JR, Evans F, Fu Y-P (2018) Re-evaluating pathogenicity of variants associated with the long QT syndrome. Journal of Cardiovascular Electrophysiology 29: 98–104. 28988457 10.1111/jce.13355 PMC5777879

[23] Walsh R, Thomson KL, Ware JS, Funke BH, Woodley J, et al. (2017) Reassessment of Mendelian gene pathogenicity using 7,855 cardiomyopathy cases and 60,706 reference samples. Genet Med 19: 192–203. doi: 10.1038/gim.2016.90 27532257 PMC5116235

[24] Berman HM, Westbrook J, Feng Z, Gilliland G, Bhat TN, et al. (2000) The Protein Data Bank. Nucleic Acids Res 28: 235–242. doi: 10.1093/nar/28.1.235 10592235 PMC102472

[25] Sievers F, Wilm A, Dineen D, Gibson TJ, Karplus K, et al. (2011) Fast, scalable generation of high-quality protein multiple sequence alignments using Clustal Omega. Molecular Systems Biology 7: 539. doi: 10.1038/msb.2011.75 21988835 PMC3261699

[26] Livingstone CD, Barton GJ (1993) Protein sequence alignments: a strategy for the hierarchical analysis of residue conservation. Computer applications in the biosciences: CABIOS 9: 745–756. 8143162 10.1093/bioinformatics/9.6.745

[27] Zvelebil MJ, Barton GJ, Taylor WR, Sternberg MJ (1987) Prediction of protein secondary structure and active sites using the alignment of homologous sequences. J Mol Biol 195: 957–961. doi: 10.1016/0022-2836(87)90501-8 3656439

[28] Golicz A, Troshin PV, Madeira F, Martin DMA, Procter JB, et al. (2018) AACon: A Fast Amino Acid Conservation Calculation Service. Submitted paper.

[29] Xu H, Li T, Rohou A, Arthur CP, Tzakoniati F, et al. (2019) Structural Basis of Nav1.7 Inhibition by a Gating-Modifier Spider Toxin. Cell 176: 1238–1239. doi: 10.1016/j.cell.2019.01.047 30794776

[30] Jiang D, Tonggu L, Gamal El-Din TM, Banh R, Pomes R, et al. (2021) Structural basis for voltage-sensor trapping of the cardiac sodium channel by a deathstalker scorpion toxin. Nat Commun 12: 128. doi: 10.1038/s41467-020-20078-3 33397917 PMC7782738

[31] Nilius B, Prenen J, Droogmans G, Voets T, Vennekens R, et al. (2003) Voltage dependence of the Ca2+-activated cation channel TRPM4. J Biol Chem 278: 30813–30820. doi: 10.1074/jbc.M305127200 12799367

[32] Zaytseva AK, Boitsov AS, Kostareva AA, Zhorov BS (2021) Possible Interactions of Extracellular Loop IVP2-S6 With Voltage-Sensing Domain III in Cardiac Sodium Channel. Front Pharmacol 12: 742508. doi: 10.3389/fphar.2021.742508 34721031 PMC8551724

[33] Tikhonov DB, Zhorov BS (2022) P-Loop Channels: Experimental Structures, and Physics-Based and Neural Networks-Based Models. Membranes (Basel) 12. doi: 10.3390/membranes12020229 35207150 PMC8876033

[34] Korkosh VS, Zaytseva AK, Kostareva AA, Zhorov BS (2021) Intersegment Contacts of Potentially Damaging Variants of Cardiac Sodium Channel. Front Pharmacol Accepted. doi: 10.3389/fphar.2021.756415 34803699 PMC8600069

[35] Toth B, Csanady L (2012) Pore collapse underlies irreversible inactivation of TRPM2 cation channel currents. Proc Natl Acad Sci U S A 109: 13440–13445. doi: 10.1073/pnas.1204702109 22847436 PMC3421201

[36] Ackerman MJ (2015) Genetic purgatory and the cardiac channelopathies: Exposing the variants of uncertain/unknown significance issue. Heart Rhythm 12: 2325–2331. doi: 10.1016/j.hrthm.2015.07.002 26144349

[37] Hoffman-Andrews L (2017) The known unknown: the challenges of genetic variants of uncertain significance in clinical practice. J Law Biosci 4: 648–657. doi: 10.1093/jlb/lsx038 29868193 PMC5965500

[38] Anderson CL, Munawar S, Reilly L, Kamp TJ, January CT, et al. (2022) How Functional Genomics Can Keep Pace With VUS Identification. Front Cardiovasc Med 9: 900431. doi: 10.3389/fcvm.2022.900431 35859585 PMC9291992

[39] Ng PC, Henikoff S (2003) SIFT: Predicting amino acid changes that affect protein function. Nucleic Acids Research 31: 3812–3814. doi: 10.1093/nar/gkg509 12824425 PMC168916

[40] Capriotti E, Fariselli P, Casadio R (2005) I-Mutant2.0: predicting stability changes upon mutation from the protein sequence or structure. Nucleic Acids Res 33: W306–310. doi: 10.1093/nar/gki375 15980478 PMC1160136

[41] Schymkowitz J, Borg J, Stricher F, Nys R, Rousseau F, et al. (2005) The FoldX web server: an online force field. Nucleic Acids Res 33: W382–388. doi: 10.1093/nar/gki387 15980494 PMC1160148

[42] Parthiban V, Gromiha MM, Schomburg D (2006) CUPSAT: prediction of protein stability upon point mutations. Nucleic Acids Res 34: W239–242. doi: 10.1093/nar/gkl190 16845001 PMC1538884

[43] Thusberg J, Olatubosun A, Vihinen M (2011) Performance of mutation pathogenicity prediction methods on missense variants. Human mutation 32: 358–368. doi: 10.1002/humu.21445 21412949

[44] Nikam R, Kulandaisamy A, Harini K, Sharma D, Gromiha MM (2021) ProThermDB: thermodynamic database for proteins and mutants revisited after 15 years. Nucleic Acids Res 49: D420–D424. doi: 10.1093/nar/gkaa1035 33196841 PMC7778892

[45] Ioannidis NM, Rothstein JH, Pejaver V, Middha S, McDonnell SK, et al. (2016) REVEL: An Ensemble Method for Predicting the Pathogenicity of Rare Missense Variants. American Journal of Human Genetics 99: 877–885. doi: 10.1016/j.ajhg.2016.08.016 27666373 PMC5065685

[46] Alirezaie N, Kernohan KD, Hartley T, Majewski J, Hocking TD (2018) ClinPred: Prediction Tool to Identify Disease-Relevant Nonsynonymous Single-Nucleotide Variants. American Journal of Human Genetics 103: 474–483. doi: 10.1016/j.ajhg.2018.08.005 30220433 PMC6174354

[47] Ware JS, Walsh R, Cunningham F, Birney E, Cook SA (2012) Paralogous annotation of disease-causing variants in long QT syndrome genes. Hum Mutat 33: 1188–1191. doi: 10.1002/humu.22114 22581653 PMC4640174

[48] Jimenez I, Prado Y, Marchant F, Otero C, Eltit F, et al. (2020) TRPM Channels in Human Diseases. Cells 9. 10.3390/cells9122604 PMC7761947 33291725

[49] Clapham DE (2003) TRP channels as cellular sensors. Nature 426: 517–524. doi: 10.1038/nature02196 14654832

[50] Nilius B, Prenen J, Janssens A, Voets T, Droogmans G (2004) Decavanadate modulates gating of TRPM4 cation channels. J Physiol 560: 753–765. doi: 10.1113/jphysiol.2004.070839 15331675 PMC1665285

[51] Xian W, Hui X, Tian Q, Wang H, Moretti A, et al. (2018) Aberrant Deactivation-Induced Gain of Function in TRPM4 Mutant Is Associated with Human Cardiac Conduction Block. Cell Rep 24: 724–731. doi: 10.1016/j.celrep.2018.06.034 30021168

[52] Boukenna M, Arullampalam P, Taib C, Guichard S, Jean-Sébastien Rougier J-S, et al. (2023) Amino acid substitution in the S1 or CH1-CH2 linker domain of TRPM4: Two new TRPM4 variants found in complete heart block patients lead to gain of expression and gain of current. bioRxiv preprint.

[53] Liu H, El Zein L, Kruse M, Guinamard R, Beckmann A, et al. (2010) Gain-of-function mutations in TRPM4 cause autosomal dominant isolated cardiac conduction disease. Circ Cardiovasc Genet 3: 374–385. doi: 10.1161/CIRCGENETICS.109.930867 20562447

